# Mirror Visual Feedback Training Improves Intermanual Transfer in a Sport-Specific Task: A Comparison between Different Skill Levels

**DOI:** 10.1155/2016/8628039

**Published:** 2016-08-24

**Authors:** Fabian Steinberg, Nils Henrik Pixa, Michael Doppelmayr

**Affiliations:** Institute of Sport Science, Department of Sport Psychology, Johannes Gutenberg University Mainz, Mainz, Germany

## Abstract

Mirror training therapy is a promising tool to initiate neural plasticity and facilitate the recovery process of motor skills after diseases such as stroke or hemiparesis by improving the intermanual transfer of fine motor skills in healthy people as well as in patients. This study evaluated whether these augmented performance improvements by mirror visual feedback (MVF) could be used for learning a sport-specific skill and if the effects are modulated by skill level. A sample of 39 young, healthy, and experienced basketball and handball players and 41 novices performed a stationary basketball dribble task at a mirror box in a standing position and received either MVF or direct feedback. After four training days using only the right hand, performance of both hands improved from pre- to posttest measurements. Only the left hand (untrained) performance of the experienced participants receiving MVF was more pronounced than for the control group. This indicates that intermanual motor transfer can be improved by MVF in a sport-specific task. However, this effect cannot be generalized to motor learning per se since it is modulated by individuals' skill level, a factor that might be considered in mirror therapy research.

## 1. Introduction

It is well known that training both limbs facilitates performance through intermanual transfer from one limb to the other. For example, the training of a specific skill with one hand can improve the performance of the other hand [1–3]. These well documented transfer effects, originally handled under the term “cross-education,” have been described for a range of fine motor skills [1, 2], sport-specific skills (e.g., [4, 5]), and muscle strength transfer [6, 7].

Several models have been put forward to explain empirical observations of intermanual transfer effects. Those theories are primarily based on studies that investigated transfer effects by the use of behavioral, neuroimaging, or brain modulation methods [6–11]. According to a recent review by Ruddy and Carson (2013) [11], two different theoretical models can be distinguished: the bilateral access (also known as the callosal access) model and the cross-activation model. The bilateral access model supports the idea that motor engrams, evolved after unilateral training in the dominant hemisphere, can be accessed by the opposite hemisphere via the corpus callosum, which leads to increased task performance of the contralateral limb [12]. The cross-activation model is supported by observations that unilateral motor executions evoke increased neuronal excitability of both the contra- and ipsilateral motor cortices, leading to neural plasticity in* both* hemispheres (cf. [8]). However, the underlying neurophysiology of bilateral transfer effects remains unclear. One possible mechanism was summarized from a recent review on neuroimaging studies in which it is argued that the mirror neural system (MNS) could be involved during bilateral transfer [7]. The MNS has been identified as the neuroanatomical basis that matches observed actions with an internal motor representation of the observed action such that the respective neuronal structures are active when movements are observed (own or others), imitated, imagined, or executed [13, 14]. Zult et al. (2014) [7] argued that imitation plays a role in motor learning during intermanual transfer paradigms, which is supported by studies observing increased activations of brain areas during cross-education that overlap with areas containing mirror neurons [15–17].

Given the positive possibilities of intermanual transfer effects, in the recent past, interest emerged regarding a method that facilitates motor learning and intermanual transfer in the clinical context, known as mirror therapy [18]. This therapy uses a mirror that is placed in the midsagittal plane of a patient in order to provide visual feedback of an intact hand that is performing a motor task while the performer directs their gaze and attention onto the mirror. Simultaneously, the nontraining hand is hidden behind the mirror in a similar position. This superimposition provides an online visual illusion as if the contralateral nontraining limb (and impaired limb in patients) is moving as efficiently as the training limb. This therapy was originally used to treat phantom limb pain [19] and is currently thought to be supportive in patients with hemiparesis [20] and complex regional pain syndrome [21] and in stroke [22, 23]. The advantage of such a method is especially valuable for patients whose control of one hand is impaired or immobilized since in motor recovery programs the additional mirror visual feedback (MVF) can facilitate the recovery process [20]. In addition, the use of this novel technique has been repeatedly demonstrated to facilitate motor learning not only in patients practicing bilaterally, but also in healthy people performing unilateral motor training. Intermanual transfer was more pronounced by the use of MVF compared to other feedback modalities [24–26].

Those studies indicate that augmented visual feedback through a mirror facilitates intermanual transfer effects, while the underlying mechanisms remain unclear. Based on neuroimaging data, a recent review by Deconinck et al. (2015) [20] found that MVF-related neural activation patterns have substantial overlap with regions related to attention and action monitoring processes, both of which are strongly related to motor learning. Additionally and in line with another review on cross-education, increased neural activity of ipsilateral brain areas that are associated with the mirror neural system was reported in mirror training [6]. Since motor execution that is concurrently observed through a mirror (i.e., providing an illusion of movement of the contralateral hand, although not active) is a special kind of movement observation, it appears to be reasonable that the MNS could be involved [6, 7]. Therefore, the involvement of the MNS-related brain areas not only is proposed to play a role in intermanual transfer but also might be synergistically involved in the augmented transfer effects supported by visual feedback through a mirror [6].

So far, most studies concerning mirror-feedback are solely based on fine motor skills, so it remains unclear whether facilitation of intermanual transfer through MVF might also occur in tasks that require more complex (and sport-specific) motor abilities. It has been repeatedly claimed that the impact of augmented feedback methods depends on task complexity and skill level [27, 28], but MVF studies did not consider whether skill level or task complexity might influence the beneficial performance gains through MVF (i.e., whether performance gains differ in terms of experienced versus unexperienced or high versus low level of expertise in a complex motor skill). In sport science and other fields of expertise research, it is well established that skill level modulates motor execution [29–31], neural activity of the action observation network [32–34], action anticipation [35], focus of attention [36], and gaze behavior [37]. Moreover, experts MNS activation is differently with higher involvement of the MNS when observed movements are familiar compared to nonfamiliar (i.e., are part of their existing motor repertoire) [38, 39].

Therefore, the present study explores whether MVF may have beneficial effects on intermanual transfer in a sport-specific task and if it is modulated by skill level. To this end, we adapted the mirror therapy test apparatus and test protocol as reported by Hamzei et al. (2012) in order to allow participants of high and low proficiency in ball dribbling to perform a dribble task while they received either visual feedback of the trained hand through a mirror (i.e., visual illusion of the left hand) or direct feedback of the trained hand. Based on the existing literature on intermanual transfer effects, for which MVF has been shown to be supportive, we hypothesized that in a more complex sport-specific task we will find intermanual transfer effects that will be increased through MVF. More specifically, due to well-known novice-expert differences and the differential activation of the MNS, we hypothesized that athletes with experience in ball dribbling will profit more than novices from MVF.

## 2. Methods

### 2.1. Participants

Initially 84 right handed participants took part in this study, but four of them were not able to complete the whole training program due to injury or other engagements. The remaining 80 participants were 24.87 ± 4.14 years old (41 females). Of the sample, 39 participants (20 females) had experience in dribbling as evidenced by actively playing either handball or basketball in a club including participation in competitions. We recruited the sample from four teams within three different German sport clubs that participated in divisions that can be classified in the midrange of the amateur level. Two teams (one female team) were two levels below the professional division and the other two (one female team) were three divisions below the professional divisions of the German division system. This expertise level could be expressed in numbers when considering that the highest division (“1. Bundesliga”) is one and the lowest eight for male and seven for female. Males had a mean expertise level of 3.9 ± 0.87, females had a mean level of 4.1 ± 0.55, and the whole sample had a mean level of 4.05 ± 0.71. The other forty-one participants (21 females) had no competition or sport club experience in sports requiring dribbling skills. However, novice participants were recruited from physical education students who are required to have at least minimal ball dribbling skills to fulfill course objectives. Given our test design (see below), minimal ball dribbling skills were needed to perform the task properly. Prior to the experiment, all participants were fully informed of the purpose of the study. The test protocol was in accordance with the Helsinki declaration and approved by the ethics committee of the Deutsche Gesellschaft für Psychologie.

### 2.2. Mirror Training and Slalom Course Setup

**Figure 1:**
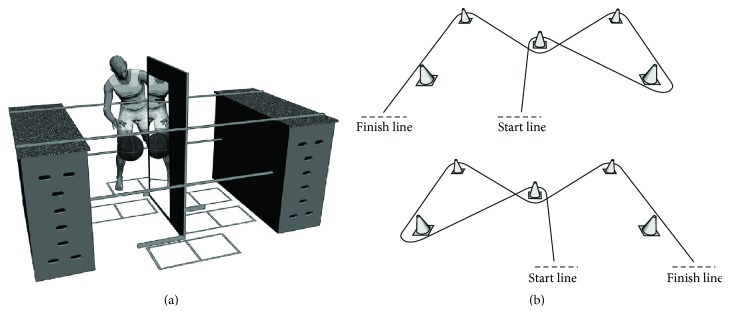
(a) The mirror box apparatus with the mirror being in the midsagittal plane of a participant; details in the text. (b) The slalom course setup for right hand dribbling (bottom) and for left hand dribbling (top).

The mirror apparatus used in the present study was based on a typical mirror therapy box (e.g., Hamzei et al. 2012) [26] and was modified for the purpose of the present study. As depicted in Figure 1(a), a 120 × 50 cm mirror was attached to a 1.70-meter wooden wall that was placed in the middle of the construction. In front of the mirror, two marked-out fields indicated the positioning of the feet, guaranteeing that the mirror was in the midsagittal line of the participant. To the left and right of the mirror, two 34 × 34 cm fields made of wooden beams served as the target fields in which a basketball could be dribbled. These fields were designed to allow three different task executions: dribbling with the right hand with visual feedback through a mirror, dribbling with the right hand without MVF (i.e., direct feedback), and dribbling with the left hand without MVF. To ensure standardized task execution and comparability between individuals, a predefined range of hand and arm motions during dribbling was defined by two ropes that were stretched between two vaulting boxes at 80 cm and 130 cm.

In addition to the mirror task, participants had to perform a dribbling slalom course (Figure 1(b)), which served as a transfer task. This course was constructed to allow participants to dribble through pylons, including five directional changes with the starting line also being the finish line. This course was constructed once for right hand dribbling (Figure 1(b), bottom) and once in a mirrored fashion for left hand dribbling (Figure 1(b), top).

### 2.3. Motor Tasks

Participants had to perform a stationary dribble task at the mirror box construction and a dynamic slalom dribble task at the slalom course. In the stationary dribble task, participants assumed a standing position with their feet placed in the respective positions (see Figure 1) and were asked to dribble a basketball (sized appropriately for their gender) with either the right or the left hand as often as possible in the two target fields in a predefined sequence, which was as follows: they were free to begin in either the left or the right field by dribbling two times in the respective field, then two times in the neighboring field, then going back again, and so on. In case of a dribbling error, which occurred when the basketball touched the wooden beams, they were instructed to proceed with dribbling and ignore the error if possible. However, in the case of a complete loss of ball control, they could take a new basketball from a box, which was directly positioned beside them, and proceed with dribbling in the respective field where the error happened. Participants' resting hand had to be positioned behind the wall at the same height and position as the other hand. The task lasted 45 seconds, and successful dribbling was defined as the ball being dribbled in a field in the instructed sequence. They were not allowed to hold the ball or to bring the hand completely under the ball. In the slalom dribbling course, the task was to dribble the ball using only one hand while running as fast as possible through the cones that were placed on the floor as depicted in Figure 1.

### 2.4. Procedure

The experiment was executed in a sports hall. After arriving and receiving instruction as to the purpose of the study, participants were pseudorandomly assigned to either the mirror visual feedback (MVF) or the control (direct feedback) group. This pseudorandomization was separated into novice and experienced (according to dribbling expertise) participants, resulting in two groups (41 novice versus 39 experienced). In the expertise group 19 participants were assigned to the MVF and 20 to the control condition, while in the novice group 20 went to MVF and 21 to the control condition. Each participant began the experiment after an individual warm-up with the dynamic slalom dribbling task. For task familiarization, verbal instructions for the slalom course were provided first and then each participant was allowed to complete the slalom course two times in a self-paced manner. In a counterbalanced order within each group, participants started the first trial with either right hand or left hand dribbling while the time for course completion was recorded via a handheld stop watch. Three trials were performed with each hand.

Subsequently, participants were instructed to the stationary dribbling task at the mirror box. For task familiarization, participants were allowed to perform the task three times (45 sec) with rest breaks of 45 sec between trials for each hand. After familiarization, baseline measurements (pretest) included three trials of 45 sec with rest breaks of 45 sec between trials for each hand performed in a counterbalanced order with the right and the left hand.

In the first training block, which followed the baseline measurements, participants performed 10 trials of 45 sec of the motor task with the right hand with rest breaks of 45 sec between each trial. In the control training condition, participants learned the motor task in the same way as was requested in the baseline measurements, with direct visual feedback of the right hand while dribbling the basketball. In the MVF training group, participants were requested to direct their gaze and attention onto the mirror while performing the motor task so that the mirror provided an illusion as if the left hand was dribbling the ball. In all, each participant performed four training blocks with the right hand as described for the first training session. The training period as well as posttest measurements were completed in a two-week interval, and only one training block was allowed per day. After the training blocks, posttest measurements were conducted on a separate day in the same way as the baseline measurements.

### 2.5. Data Analysis

Pre- and posttest trials were recorded with an HD video camera directed at the target fields for subsequent analysis of scores and errors. An investigator blinded to expertise level analyzed the video material and assigned scores as defined above (i.e., one point was given for successfully dribbling in a target field). Moreover, the error score was counted as well, such as a loss of ball control. Thus, dribbling scores (further called “dribbling performance”) for the three trials with their respective errors (further called “dribbling error”) could be analyzed for the pre- and the posttest and for each hand. Likewise, the three scores of the three trials were measured for the pre- and posttests of the slalom course (further called “slalom performance”) for both hands. Statistical analysis presented here was performed by taking the mean values of the three pre- and posttest trials. We took the mean values since we could not find any statistical differences when we performed the same analysis using the median score or the best score.

### 2.6. Statistical Analysis

Shapiro-Wilks tests were used to check all variables for normal distribution; all scores of the stationary dribbling at the mirror box (i.e., dribbling performance) were normally distributed. However, error scores of the dribbling task at the mirror box (i.e., dribbling error) as well as times for the slalom course (i.e., slalom performance) were not normally distributed. Thus, ANOVAs and *t*-tests were used for normally distributed variables (i.e., only for the dribbling performance at the mirror test setup for the right and the left hand), while for the others Mann-Whitney *U* test were computed (see below). Before result presentation of the training effects, a section (statistics on baseline performance) is included to check for baseline differences between conditions (MVF versus control) by calculating independent *t*-tests or *U*-tests for each variable separated for each expertise level (i.e., within the experienced and within the novice group). Moreover, we compared the baseline scores of our parameters (dribbling performance, dribbling error, and slalom performance) by independent *t*-tests or *U*-test between experienced and novice group to check if pretest values mirror our group delineation (i.e., novices versus experienced participants). Effect sizes of *t*-test were estimated as Cohen's *d*, where *d* > 0.2 indicates a small effect, *d* > 0.05 a medium effect, and *d* > 0.8 a large effect [40]. Effect sizes based on *z*-scores computed by the *U*-tests were estimated by Pearson's correlation coefficients, where *r* > 0.1 indicates a small effect, *r* > 0.3 a medium effect, and *r* > 0.5 a large effect [40].

Three-way ANOVAs with repeated measures “TIME” (Pre/Post) on the between-factor “CONDITION” (MVF/Control) and “EXPERTISE” (Novice/Experienced) were calculated separately for left and right hand performance to observe whether the feedback modalities (MVF versus active) influence a different performance improvement in the two expertise groups. Wherever sphericity was violated, Greenhouse-Geisser adjusted values were reported and *p* values below the 5% thresholds were considered statistically significant. Effect sizes of ANOVAs were estimated as partial eta-squares (*η*_*p*_^2^), where *η*_*p*_^2^ > 0.01 indicates a small, *η*_*p*_^2^ > 0.06 a medium, and *η*_*p*_^2^ > 0.14 a large effect [40], and these were reported whenever significance dropped below 5%.

Finally, for the not-normally distributed variables (dribbling error and slalom performance), an index for performance changes was calculated by subtracting post- from prevalues. For these variables, between-subject performances changes between the MVF and control groups were analyzed by Mann-Whitney *U* tests separately for novice and experienced participants.

### 2.7. Statistics on Baseline Performance

Since in the following analysis several between-subject analyses were performed and body height might influence dribbling behavior at a test setup not adjusted to body height, we calculated ANOVAs with the between-factor CONDITION and EXPERTISE for body height to determine whether experimental groups systematically differ in these variables. However, no effects emerged (all *p* > 0.05). *t*-tests (for dribbling performance) and *U*-tests (for dribbling error and slalom performance) for baseline (pretest) scores revealed that, for all three test variables (i.e., left and right hand dribbling performance, dribbling error, and slalom performance), no significant difference between control and MVF (all *p* > 0.05) within the novice group or between control and MVF of the experienced group (all *p* > 0.05) emerged. This indicates that the control groups compared to the MVF groups did not start at different performance levels and thus different learning rates cannot be attributed to different baseline values.

We additionally tested whether our a priori group delineation criteria (active engagement in basketball or handball competition) were able to separate experienced from novice participants, that is, if it is mirrored in different baseline measures of our three outcome variables: Table 1 (values separated by the factor EXPERTISE) depicts the values of baseline (pretest) left and right hand performance from which one can see that left hand (*t*(78) = 7.63; *p* < 0.001; *d* = 1.70) as well as right hand (*t*(78) = 5.14; *p* < 0.001; *d* = 1.15) dribbling performance of the experienced participants was significantly higher than that of novices with high effect sizes. *U*-test revealed that (not-normally distributed variables) this pattern was mirrored in different dribbling error scores in the left hand (*z* = −2.82; *p* < 0.01; *r* = −0.31) and right hand (*z* = −2.58; *p* < 0.05; *r* = −0.28). Finally, we found expertise differences in the time for slalom performance in the left hand (*z* = −2.63; *p* < 0.01; *r* = −0.29) as well as in the right hand (*z* = −2.00; *p* < 0.05; *r* = −0.22) with better scores for the experience group compared to the novice group. These differences indicate that our delineation criteria were successful in separating novices from experienced participants for our sport-specific motor tasks.

## 3. Results

### 3.1. Global Analysis on the Central Parameter Dribbling Performance

As expected after four training sessions, a three-way ANOVA for the right hand dribbling performance revealed a highly significant performance improvement for the factor TIME (*F*(1,76) = 178.31; *p* < 0.001; *η*_*p*_^2^ = 0.701), while all other factors and their interactions remained nonsignificant (all *p* > 0.05). Thus, right hand performance improvements did not differ whether the right hand was trained at a mirror or whether the participant was novice or experienced. A three-way ANOVA for the parameter left hand dribbling performance revealed significant main and interaction effects. There was a highly significant effect for the factor TIME, meaning performance improved from pre- to posttest measurements (*F*(1,76) = 175.37; *p* < 0.001; *η*_*p*_^2^ = 0.698). A medium, significant effect was found for the interaction TIME*∗*CONDITION (*F*(1,76) = 5.09; *p* < 0.05; *η*_*p*_^2^ = 0.063). Another small but significant two-way interaction effect appeared between the factors TIME*∗*EXPERTISE (*F*(1,76) = 4.32; *p* < 0.05; *η*_*p*_^2^ = 0.054). Finally, a significant threefold interaction of TIME*∗*CONDITION*∗*EXPERTISE was noted (*F*(1,76) = 7.27; *p* < 0.01; *η*_*p*_^2^ = 0.087), indicating that performance improvements from pre- to posttests were differently effected by the kind of training (i.e., MVF versus active feedback training) and by the expertise level (i.e., experts versus novices). Since our participant group consisted of male and female participants that were almost evenly distributed across groups we exploratively included GENDER (male/female) as an additional factor in the ANOVA model but could not detect any significant GENDER effects (all *p* > 0.05).

### 3.2. Left Hand Dribbling Performance Improvements between Conditions

**Figure 2:**
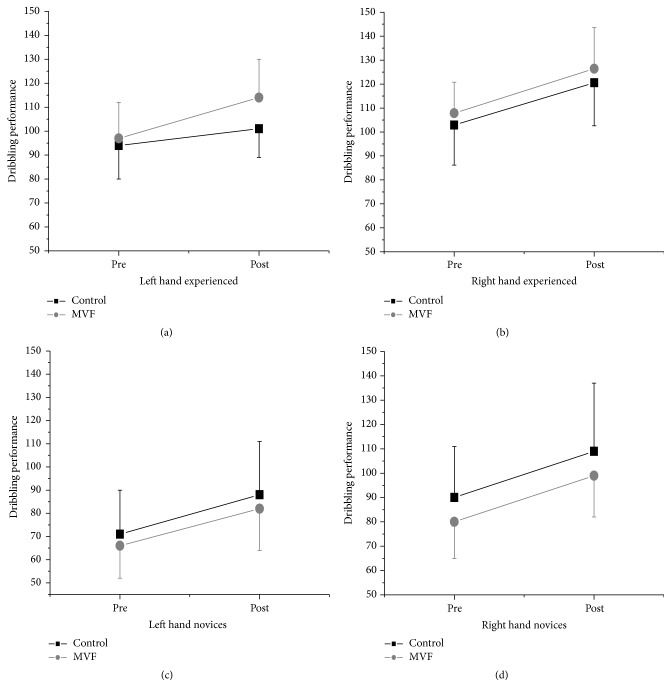
Interaction plots for the main outcome variable (dribbling performance) separated for novice and experienced groups and for left and right hand. Mean absolute scores are depicted and error bars indicate standard deviations; statistics are explained in the text.

Due to the twofold and threefold interactions for left hand performance, we performed separate ANOVAs, one for the novice and one for the experienced group (since no interaction effects for right hand appeared, no further analyses were performed for right hand; see data analysis). As depicted in Figure 2(c), for the novice group a significant improvement for the factor TIME in left hand performance emerged (*F*(1,39) = 96.48; *p* < 0.001; *η*_*p*_^2^ = 0.712), but the interaction between TIME*∗*CONDITION was not significant (*p* > 0.05, see Figure 2(c)). A reversed pattern emerged for the experienced group in left hand performance improvement. As shown in Figure 2(a), the ANOVA for left hand dribbling indicated a significant effect for the factor TIME (*F*(1,37) = 82.30; *p* < 0.001; *η*_*p*_^2^ = 0.690) and a highly significant TIME*∗*CONDITION effect (*F*(1,37) = 16.21; *p* < 0.001; *η*_*p*_^2^ = 0.305). Although there were no interaction effects for right hand dribbling, performance measures for this hand are depicted in Figure 2 as well (Figures 2(b) and 2(d)) to account for the significant TIME effects.

### 3.3. Post Hoc Measures for Left Hand Dribbling Performance Improvements

**Figure 3:**
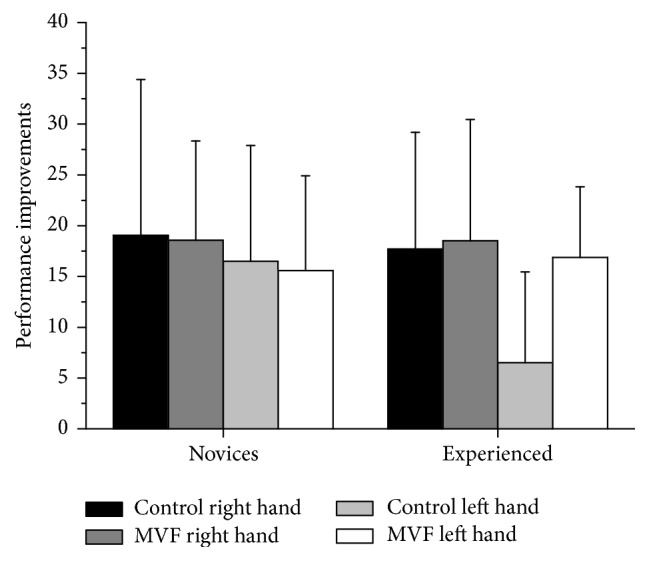
Shown are the performance improvements for the main outcome variable (dribbling performance) calculated by subtracting prevalues from postvalues and separated for novice and experienced groups. Scores depicted are absolute means, and error bars indicate standard deviations. The higher the value, the greater the improvement; statistics are explained in the text.

For post hoc analysis to further elucidate the significant TIME *∗* CONDITION and TIME *∗* CONDITION *∗* EXPERTISE effects, we calculated a performance improvement score (posttest minus pretest). As clearly indicated by Figure 3 and relative improvements (in %) depicted in Table 2, Bonferroni-corrected independent *t*-tests revealed that only left hand performance gains in the experienced group significantly differed between MVF and control group (*t*(37) = −4.02; *p* < 0.01; *d* = −1.29), with higher improvements for the MVF group. Novices' performance improvements did not differ significantly between control and MVF (*p* > 0.05). Moreover, the novice control group improved significantly more than the control group of experienced participants (*t*(39) = −3.11; *p* < 0.05; *d* = −0.97), while the MVF groups did not differ significantly (*p* > 0.05). For a complete overview of test results, right hand performance improvements are shown in Figure 3.

### 3.4. Dribbling Error Improvements between Groups

**Figure 4:**
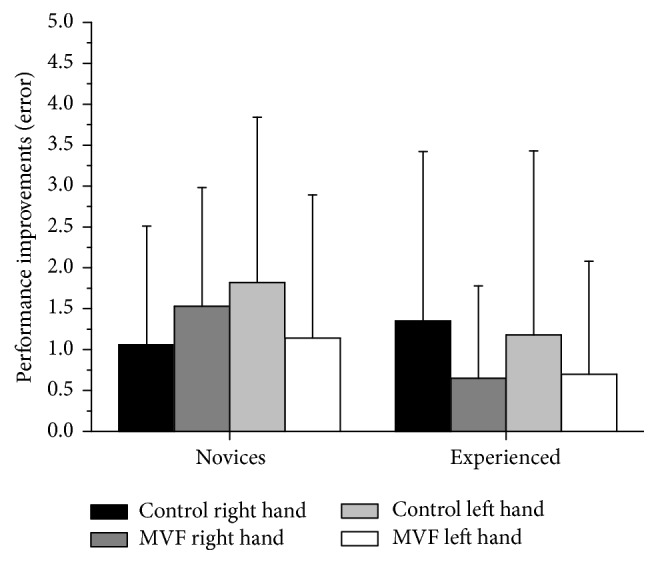
Shown are the performance improvements for the dribbling error, which was assessed for the dribbling task performed at the mirror box. Error improvements were calculated by subtracting prevalues from postvalues and are depicted separately for novice and experienced groups. Illustrated scores are absolute means, and error bars indicate standard deviations. Note that a positive value indicates improvement; statistics are explained in the text.

To observe whether the MVF group's dribbling error decrease from pre- to posttest measures (i.e., performance improvements) differed from the control groups, we compared the difference in the pre- and posttest measures separated for the factors CONDITION*∗*EXPERTISE. Table 2 depicts absolute and relative error decreases (%) of all dependent measures, while Figure 4 shows only the error decrease (note that a positive value indicates error decrease, not increase). However, for the right and left hand, error decreases (i.e., error improvements) did not differ between MVF and control (both *p* > 0.05) or for novices and experienced participants (both *p* > 0.05); thus the dribbling error decrease was constant across experimental groups.

### 3.5. Comparison of Slalom Performance Improvements between Groups

**Figure 5:**
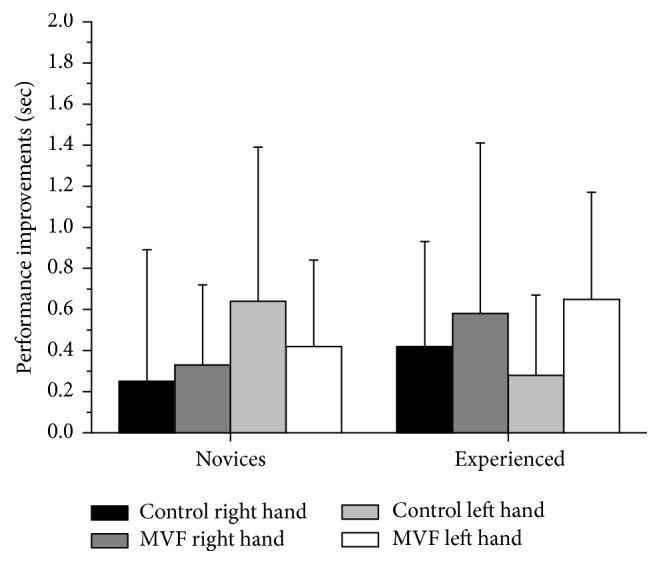
Shown are the performance improvements in the time for slalom dribbling parkour execution (i.e., slalom performance) calculated by subtracting prevalues from postvalues and separated for novice and experienced groups. These scores are absolute means, and error bars indicate standard deviations. The higher the value, the greater the improvement; statistics are explained in the text.

To detect whether the MVF group's performance improvements in slalom dribbling were more pronounced than the control groups, we compared the difference scores from pre- to posttest measures separated for the factors CONDITION*∗*EXPERTISE. Since the slalom dribbling performance scores were not normally distributed, instead of ANOVAs, nonparametric tests were computed (*U*-tests; see statistic section) and *r*-values indicate effects size estimations based on *z*-values [40]. Table 2 depicts absolute and relative performance improvements (%) of all dependent measures; Figure 5 shows only the slalom dribbling performance improvements.

We found no differences between MVF and control in right hand improvements, neither for experienced nor for novice participants (both *p* > 0.05). However, for left hand performance, comparison of the improvements between MVF and control in the experienced group yielded significance, with the MVF group improvement greater than that of the control group (*z* = −2.529; *p* < 0.05; *r* = −0.58), while the novice MVF group did not significantly improve performance with the left hand (*p* > 0.05).

## 4. Discussion 

The purpose of the present approach was to find out whether mirror visual feedback is applicable within a sport-scientific context and whether the well-known improvements of motor learning through MVF are modulated by skill level. Intermanual transfer effects were found, such as all groups, regardless of proficiency level or feedback modality, improving performance with the nontraining hand. Compared to direct feedback, superior left hand performance gains of MVF participants were significant in the experienced group only, while performance gains in novice participants did not differ between feedback modalities. Interestingly, this pattern of observations was accompanied by the same effects in a (nontrained) transfer task in which dynamic instead of stationary dribbling was requested. The results support our first hypothesis, which predicted intermanual transfer effects in the present motor task, which will be increased through MVF. However, this hypothesis has to be modified, as MVF effects depend on skill level. Consequently, our second hypothesis that experts will profit more from MVF than novices has to be rejected in its current formulation, since only experienced players showed improvement. A comparable decrease in error scores across groups indicates that a change in the speed and accuracy relationship cannot account for our findings. Moreover, we could not find any differences between male or female participants, which, however, should be handled cautiously due to the limited participant number for each gender and factor.

### 4.1. Intermanual Transfer

The intermanual transfer effects observed in the present study are in line with other studies that observed transfer effects of different motor tasks. For example, transfer effects were found in several realistic sport-specific tasks [4, 5, 41], a pegboard task requiring fine motor skills [2], an inverted-reversed printing task [42], finger tapping [43], keyboard pressing [12], ball catching [44], and adaptation in visuomotor rotation tasks [45]. Thus, the present study extends those findings in so far that interlimb transfer effects are observable in a ball dribbling task, which, however, are modulated by skill level.

Interestingly, transfer effects of the control groups receiving direct feedback were stronger in novice participants compared to those who were experienced. However, ball dribbling is a well-learned motor behavior for handball and basketball players, but not for nonplayers. It is well-known that motor learning undergoes several stages [46] such that the task to be executed in the present study differed in several aspects with respect to skill level. It is thought that, during the initial motor learning phase, movements are unskilled and depend strongly on feedback along with high demands on attention [47, 48]. With sustained practice, movement aspects such as accuracy and velocity increase and become more automated, while dependence on feedback becomes less important [49]. Since experienced participants might have been less dependent on feedback, one could assume that the level of attention decreased in the direct feedback modality, which in turn resulted in a lesser degree of intermanual transfer effects to the left hand compared to novice participants. However, this is a speculative view which needs further investigation of the explicit role of attention in intermanual transfer.

### 4.2. Mirror Visual Feedback

The present results concerning MVF are twofold since intermanual transfer effects were more pronounced through MVF compared to normal feedback only in the experienced group. Novice participants, relatively unfamiliar with basketball dribbling, did not benefit more from mirror visual feedback compared to normal (direct) feedback. The latter finding is in contrast to studies that found pronounced transfer effects in healthy participants (for an overview see [20]). However, due to study protocol and feedback modalities, only the studies by Hamzei et al. [26] and Läppchen et al. [25] are directly comparable to the present approach. They compared learning simple fine motor tasks (e.g., a pegboard task) with either direct feedback from the training hand or MVF. Clear advantages of MVF over direct feedback were found in intermanual transfer from right to left hand. Based on accompanied functional MRI data Hamzei et al. (2012) found a mirror training specific neural network, including areas that are associated with the mirror neural system. Moreover, Läppchen et al. (2012) found different excitability changes (induced by TMS) in M1 in both hemispheres after mirror training. The M1_left_ (contralateral to the trained hand) of the direct feedback group had increased excitability and the mirror training group had decreased M1_left_ excitability [25]. Such differential neural networks that have substantial overlap with the MNS might be one reason for augmented transfer effects compared to normal feedback [20].

Therefore, from a neurophysiological point of view, different involvement of MNS-related brain regions might be responsible for the skill-level dependent results in the current experiment. The tasks used by [25, 26] were basic fine motor tasks that were comparable to fine motor executions that humans perform in everyday life such as putting a peg in a hole or using a teaspoon. Consequently, it might well be that those tasks become or were already familiar within the training process and had become part of the motor repertoire. Indeed, Hamzei et al. (2012) argued that observation of this embodied action (i.e., the tasks used in their study) activates MNS-related brain regions, likely due to motor simulation [26]. However, the ball dribbling task in the present study has no equivalence to the everyday life of nonplayers. Therefore, although speculative and not measured directly, one might argue that the ball dribbling task, being relatively unfamiliar for novice participants, did not activate the MNS to such an extent that it drives augmented transfer effects as suggested through MVF. In contrast, experienced ball dribblers, whose sport frequently requires ball dribbling skill, might had stronger involvement of MNS-related brain regions. Support for this interpretation comes from (motor-) expert studies. In expert dancers, as an example, different activations of the MNS with higher involvement of the MNS have been detected when observed movements are familiar, that is, part of the own motor repertoire [38, 39]. Along with the activation of ipsilateral motor areas through observing the right training hand in a mirror (i.e., illusion of a moving left hand) in accordance with the cross-activation model (cf. [8]), this resulted in performance gains of the left untrained hand which outweighed any performance gains through “common” intermanual transfer effects [6, 7].

Alternatively, considering the different task demands of ball dribbling concurrently with MVF or active feedback, it could well be that task complexity plays an essential role for our findings, which in turn is related to individual skill level [50, 51]. Thus, a simple explanation could be that for experienced players the active feedback condition was simple, while MVF was complex (or at least very unusual or more complex). In contrast, both feedback modalities were complex for novice participants. The unusual feedback through a mirror might have forced even experienced ball dribblers to direct attention more strongly back to task execution as visual feedback dependency becomes more relevant. Indeed, it has been indicated that with increasing task complexity the profit of concurrent feedback also increases [50]. The increase in attention of experienced players to task execution and thus to the illusion of the left hand might have, in accordance with the cross-activation model, increased neural involvement of ipsilateral brain areas, which in turn evoked the performance improvements of the left hand. Bearing this in mind, our results are well in line with the current knowledge and further suggest that MVF-induced transfer effects could depend on the two interrelated factors' task complexity and the individual's skill level [27, 52]. If so, mirror therapy studies in the future might consider these aspects to find the best means of motor rehabilitation.

Although the present exploratory approach requires further investigation into the role of task complexity, the role of attention, skill level, and additional comparisons to other feedback modalities, we propose that the present results support MVF as being a potential tool to support intermanual transfer effects for rehabilitation in a sport context when athletes suffer hand or arm immobilization. However, considering that the present approach is the first that attempted to transfer the mirror illusion paradigm to the sport context, the study has some limitations. First, no measurement of neural activity was implemented, so the interpretations of brain-related mechanisms are only indirect and warrant further investigation by concurrent neuroimaging techniques. Furthermore, comparisons to other feedback conditions such as purely observational feedback of a passive hand [53] or active left hand, motor imagery, and bilateral training or left handed participants have not been considered in this initial study. How far professional or high expertise players are differently affected by MVF compared to the medium expertise levels in this study would be an interesting point as well. In this line, a higher sample size including a high variance of skill levels might also reveal a possible relationship between baseline values and intermanual performance gains through MVF. Lastly, proprioceptive sensations from the hand behind the mirror when unintentionally moved might have interfered with the mirror illusion differently in the two expertise groups [54], a factor that should be systematically controlled in the future.

## 5. Conclusion

The present study found that mirror visual feedback facilitates intermanual transfer effects in sport, but only for participants that had experience with the movements being performed. Thus, this study introduced the role of skill level and task complexity to the field of mirror visual feedback, two interrelated factors that could provide new insights in the study of mechanisms underlying MVF.

## Figures and Tables

**Table 1:** Performance scores for all test variables for experts and novices independent of condition. Shown are mean values along standard deviations.

Expertise level	Task/variable	Pretest		Posttest	
		Right hand	Left hand	Right hand	Left hand
Novice	Dribbling performance	85.52 ± 18.76	68.78 ± 16.63	104.33 ± 23.31	84.83 ± 20.98
Experienced		105.32 ± 15.39	95.60 ± 14.67	123.42 ± 18.07	107.15 ± 15.87

Novice	Dribbling error	2.52 ± 1.79	4.59 ± 2.74	1.23 ± 1.11	3.09 ± 2.30
Experienced		1.66 ± 1.84	2.99 ± 2.46	0.80 ± 0.93	1.93 ± 1.39

Novice	Slalom performance (sec)	9.09 ± 1.52	9.58 ± 1.69	8.80 ± 1.45	9.04 ± 1.57
Experienced		8.47 ± 1.01	8.61 ± 1.13	8.01 ± 0.87	8.11 ± 0.86

**Table 2:** Absolute and relative performance improvements for all test variables separated by CONDITION *∗* EXPERTISE. Shown are mean values along standard deviations.

CONDITION *∗* EXPERTISE	Task/variable	Performance improvements		Performance improvements^*∗*^ (%)	
		Right hand	Left hand	Right hand	Left hand
Control novice	Dribbling performance	19.06 ± 15.33	16.50 ± 11.41	17.23	11.80
MVF novice		18.55 ± 9.79	15.58 ± 9.33	14.91	10.27
Control experienced		17.70 ± 11.48	6.50 ± 8.94	18.21	6.11
MVF experienced		18.51 ± 11.93	16.87 ± 11.93	19.97	16.41

Control novice	Dribbling error	−1.06 ± 1.45	−1.82 ± 2.02	40.66	40.80
MVF novice		−1.53 ± 1.45	−1.14 ± 1.75	42.99	24.35
Control experienced		−1.35 ± 2.07	−1.18 ± 2.25	63.81	35.91
MVF experienced		−0.65 ± 1.13	−0.70 ± 1.38	37.68	28.61

Control novice	Slalom performance (sec)	−0.25 ± 0.64	−0.64 ± 0.75	6.68	2.61
MVF novice		−0.33 ± 0.39	−0.42 ± 0.42	4.43	3.63
Control experienced		−0.42 ± 0.51	−0.28 ± 0.39	3.41	4.93
MVF experienced		−0.58 ± 0.83	−0.65 ± 0.52	7.53	6.90

^*∗*^Note that the relative improvements, for example, 63.81% for the experienced control group (right hand), appear to be very high. Participants' error scores were generally relatively low, so reducing error from two errors in the pretest up to only one error in the posttest is already a 100% change.
